# Thorough QT/QTc study to evaluate the effect of a single supratherapeutic dose of islatravir on QTc interval prolongation in healthy adults

**DOI:** 10.1128/aac.00464-24

**Published:** 2024-07-02

**Authors:** Randolph P. Matthews, Yang Liu, Catherine Matthews, Kristin L. Butterfield, Terry O'Reilly, S. Aubrey Stoch, Marian Iwamoto

**Affiliations:** 1 Translational Medicine, Biostatistics and Research Decision Sciences, Merck & Co., Inc., Rahway, New Jersey, USA; 2 Clinical Research, Celerion, Tempe, Arizona, USA; Providence Portland Medical Center, Portland, Oregon, USA

**Keywords:** human immunodeficiency virus, nucleoside reverse transcriptase translocation inhibitor, islatravir, QT interval, cardiac repolarization

## Abstract

Islatravir is a deoxynucleoside analog being developed for the treatment of HIV-1 infection. Clinical studies are being conducted to evaluate islatravir, administered in combination with other antiretroviral therapies, at doses of 0.25 mg once daily and 2 mg once weekly. In multiple previous clinical studies, islatravir was generally well tolerated, with no clear trend in cardiac adverse events. A trial was conducted to evaluate the effect of islatravir on cardiac repolarization. A randomized, double-blind, active- and placebo-controlled phase 1 trial was conducted, in which a single dose of islatravir 0.75 mg, islatravir 240 mg (supratherapeutic dose), moxifloxacin 400 mg (active control), or placebo was administered. Continuous 12-lead electrocardiogram monitoring was performed before dosing through 24 hours after dosing. QT interval measurements were collected, and safety and pharmacokinetics were evaluated. Sixty-three participants were enrolled, and 59 completed the study. Fridericia’s QT correction for heart rate was inadequate; therefore, a population-specific correction was applied (QTcP). The placebo-corrected change from baseline in QTcP (ΔΔQTcP) interval at the observed geometric mean maximum plasma concentration associated with islatravir 0.75 mg and islatravir 240 mg was <10 ms at all time points. Assay sensitivity was confirmed because the use of moxifloxacin 400 mg led to a ΔΔQTcP >10 ms. The pharmacokinetic profile of islatravir was consistent with that of previous studies, and islatravir was generally well tolerated. Results from the current trial suggest that single doses of islatravir as high as 240 mg do not lead to QTc interval prolongation.

## INTRODUCTION

Human immunodeficiency virus (HIV) remains a global public health concern, with approximately 39.0 million people infected and approximately 1.3 million new infections worldwide in 2022 (1). The ongoing epidemic has resulted in a growing and collective burden on care, particularly because HIV-1 infection is a chronic condition that necessitates lifelong antiretroviral treatment (2). Islatravir is a potent inhibitor of HIV-1 reverse transcriptase (3). Unlike other nucleoside reverse transcriptase inhibitors, islatravir inhibits reverse transcription via multiple mechanisms, leading to both immediate and delayed viral DNA chain termination (4, 5). The pharmacokinetic (PK) profile of islatravir has been characterized in previous studies (6–9), in which dose-proportional PK was observed over a wide range of doses (0.25–400 mg) (7, 8). After oral administration, islatravir is rapidly absorbed; maximum plasma concentration is reached within 30–60 minutes (7, 8). Islatravir is primarily cleared from plasma by renal excretion of the unchanged parent drug and adenosine deaminase–mediated metabolism to 4′-ethynyl-2-fluoro-2′deoxyinosine (M4) (9). Islatravir is taken up into peripheral blood mononuclear cells, where it is converted to its pharmacologically active triphosphate form via endogenous intracellular kinases (3). Active islatravir triphosphate persists for long periods intracellularly, with an apparent terminal half-life of 177–204 hours, allowing for extended-interval dosing (7, 8).

Islatravir is being evaluated clinically for the treatment of HIV-1 infection as part of oral once-daily and once-weekly regimens at doses of 0.25 and 2 mg, respectively. Islatravir has been generally well tolerated in clinical studies to date. However, after a longer duration of islatravir administration at higher doses (0.75 mg daily, 20 mg weekly, and 60 mg monthly), decreases in total lymphocyte and CD4^+^ T-cell counts were observed in some participants (10).

A crucial step in the development of novel drugs is to determine cardiac safety, including the propensity of a drug to cause cardiac arrhythmias through delayed repolarization (11). Delayed repolarization can increase the risk of life-threatening cardiac arrhythmia, such as torsade de pointes (11). Results from regulatory-enabling *in vitro* human ether-à-go-go–related gene and nonhuman primate electrocardiogram (ECG) telemetry studies suggest that islatravir has a low risk of delaying cardiac repolarization in humans. Specifically, the islatravir *in vitro* human ether-à-go-go–related gene half-maximal inhibitory concentration was ≥60,000 times and ≥8,559 times more than the projected maximum plasma concentrations for daily and weekly dosing, respectively. In addition, there was no effect on corrected QT (QTc) in monkey telemetry studies at a projected maximum exposure approximately 7,200 times and 1,028 times above the maximum plasma concentration (*C*_max_) for daily and weekly dosing, respectively (Merck & Co., Inc., Rahway, NJ, USA; data on file).

To rule out the potential for QT prolongation with the use of islatravir, a thorough QT study was conducted to evaluate the effect of islatravir on the QT interval. At the time of the thorough QT study, islatravir was being assessed in clinical trials at a daily dose of 0.75 mg and a monthly dose of 60 mg, and the study was designed to assess both the lower dose (0.75 mg) and a dose supratherapeutic to 60 mg (240 mg). Although these doses were selected to cover the original daily therapeutic dose and provide a margin above the original monthly dose, a lack of an effect on QTc at 0.75 and 240 mg would also support a lack of an effect at 0.25 and 2 mg.

## RESULTS

### Study population

A total of 63 participants were randomized and received at least one dose of the study drug. For sequences 1 and 2, 26 of 28 participants (92.9%) received all doses and completed the study. For sequence 3, all seven participants (100.0%) completed the study, whereas in sequence 4, 26 of 28 participants (92.9%) received all doses and completed the study. All four study discontinuations (*n* = 2 in sequence 1 and *n* = 2 in sequence 4) were due to severe acute respiratory syndrome–related coronavirus strain 2 positivity (*n* = 3) or coronavirus disease 2019 infection (*n* = 1). The majority of participants were female (76.2%), White (88.9%), and Hispanic or Latino (77.8%), and the median age of the study participants was 41 years (range, 19–64 years) (Table 1).

**TABLE 1:** ** **Baseline characteristics and demographics of the study population^*a*^^,*b*^

	Sequence 1 (*n* = 14)	Sequence 2 (*n* = 14)	Sequence 3 (*n* = 7)	Sequence 4 (*n* = 28)	Total (*N* = 63)
Sex, *n* (%)					
Male	4 (28.6)	2 (14.3)	2 (28.6)	7 (25.0)	15 (23.8)
Female	10 (71.4)	12 (85.7)	5 (71.4)	21 (75.0)	48 (76.2)
Age, median (range) (years)	36.0 (25–58)	44.5 (19–61)	48.0 (36–61)	37.0 (20–64)	41.0 (19–64)
Race, *n* (%)					
Black or African American	1 (7.1)	1 (7.1)	1 (14.3)	3 (10.7)	6 (9.5)
Multiple	1 (7.1)	0	0	0	1 (1.6)
White	12 (85.7)	13 (92.9)	6 (85.7)	25 (89.3)	56 (88.9)
Ethnicity, *n* (%)					
Hispanic/Latino	12 (85.7)	10 (71.4)	5 (71.4)	22 (78.6)	49 (77.8)
Not Hispanic/Latino	2 (14.3)	4 (28.6)	2 (28.6)	6 (21.4)	14 (22.2)
BMI, median (range) (kg/m^2^)	26.0 (22.5–30.9)	28.7 (21.0–32.4)	27.4 (22.5–31.6)	28.4 (22.8–32.5)	27.8 (21.0–32.5)

^*a*^ Refer to Fig. 1 for the definition of sequences.

^*b*^ BMI, body mass index.

**Fig 1:**
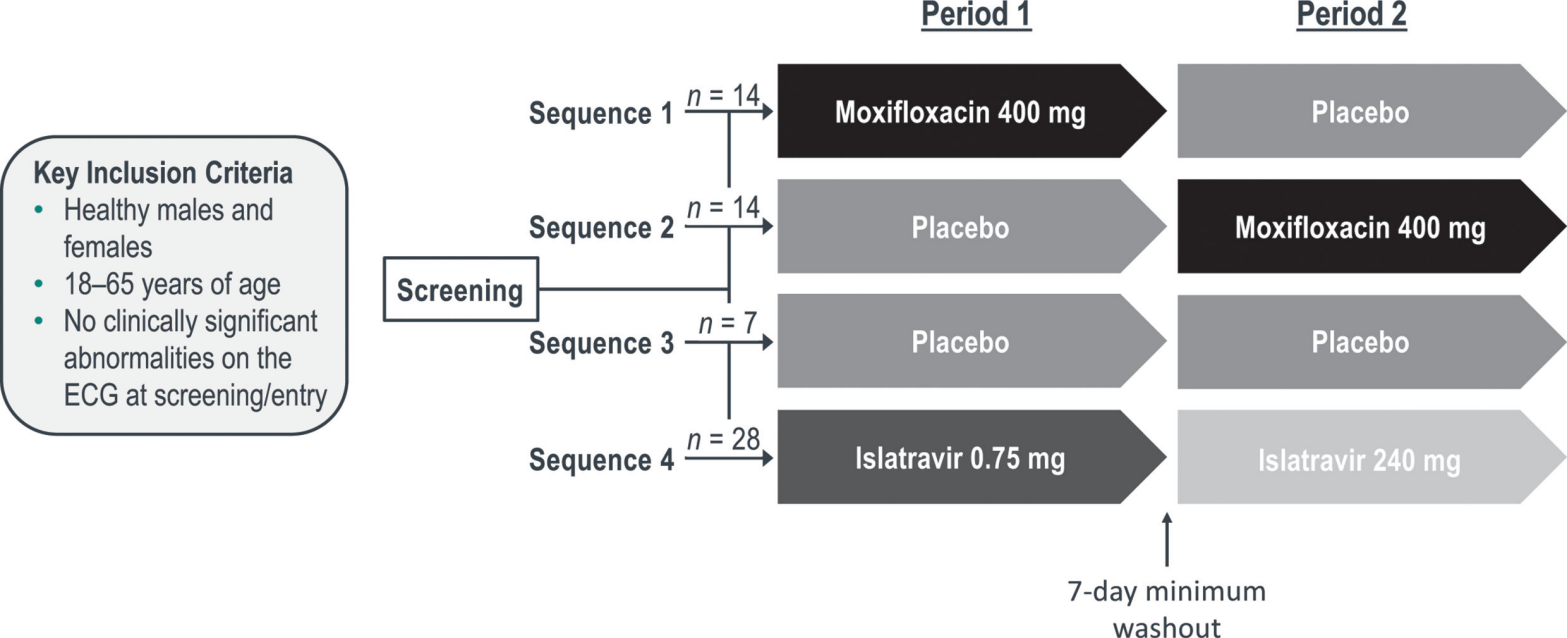
** **Study design. In crossover sequences 1 and 2, participants received either moxifloxacin 400 mg or placebo to moxifloxacin, alongside a placebo to islatravir (both 0.75 and 240 mg placebos in both periods). In sequence 3, participants received placebos to islatravir 0.75 mg, islatravir 240 mg, and moxifloxacin in both periods. In period 1 of sequence 4, participants received a single dose of islatravir 0.75 mg in addition to placebos to islatravir 240 mg and moxifloxacin; in period 2, participants received a single dose of islatravir 240 mg in addition to placebos to islatravir 0.75 mg and moxifloxacin. ECG, electrocardiogram.

### Adequacy of QT correction factor

**Fig 2:**
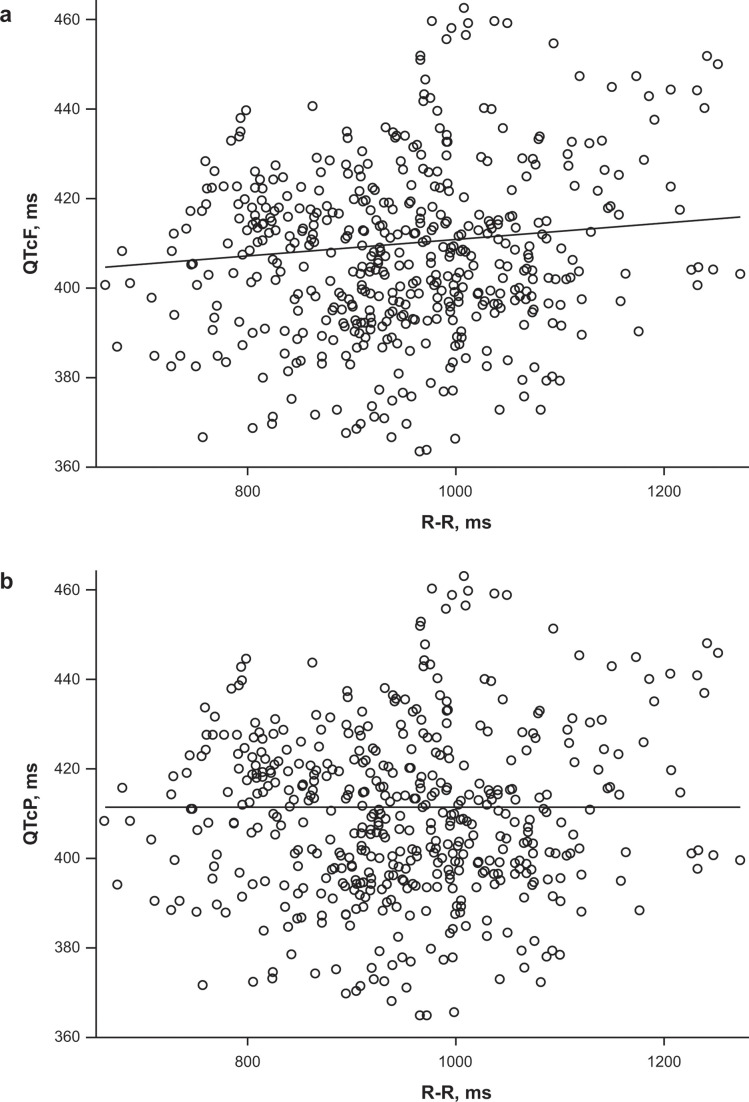
Individual (**a**) QTcF (in milliseconds) and (**b**) QTcP (in milliseconds) versus R-R (in milliseconds) values obtained during placebo and drug-free time points with estimated linear regression line. QTcF, Fridericia’s formula-corrected QT interval; QTcP, population-corrected QT interval; R-R, R wave to R wave.

Corrected QT interval using Fridericia’s correction was deemed to be an inadequate correction factor based on the regression of QTcF versus R wave to R wave (R-R) based on placebo and before dosing data because the 95% confidence interval (CI) for the estimated slope did not contain zero (Fig. 2a). However, the regression of individual QTcP versus R-R produced a slope estimate of −0.000019, with a 95% CI of the slope containing zero, showing that the QTcP correction was adequate (Fig. 2b). The QTcP correction factor was 0.3754 and was used for all subsequent analyses.

### QTc and other ECG assessments

**Fig 3:**
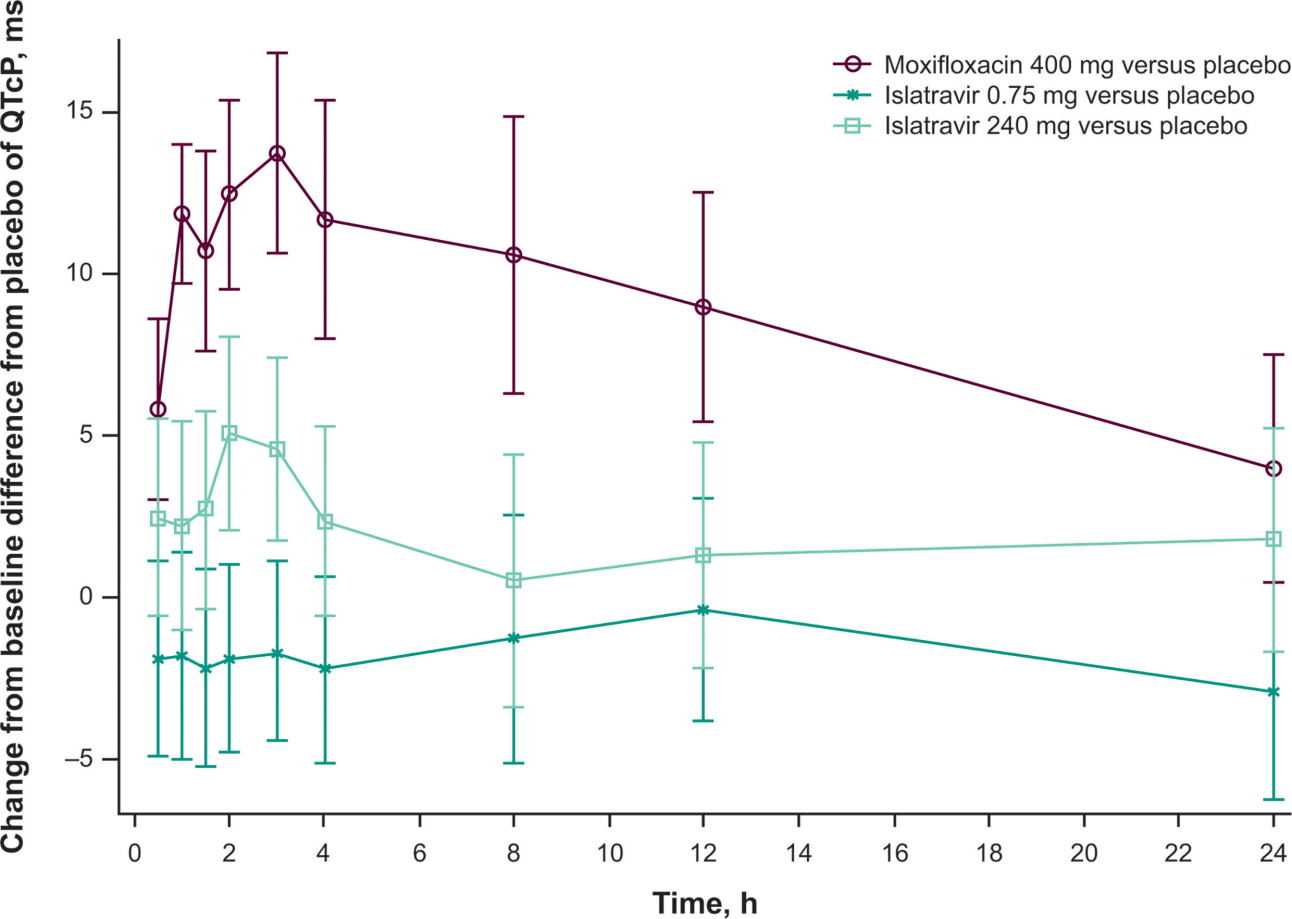
QTcP change from baseline difference from placebo (LSM difference with 90% CI) by time point and treatment. A supportive model-based time point-by-time point analysis was conducted for islatravir and moxifloxacin using a linear mixed-effects model to estimate the mean between-treatment difference (moxifloxacin–placebo and islatravir–placebo) in QTcP change from baseline at each dose level and each time point. The LSM (95% CI) of QTcP change from baseline and the least squares difference (90% CI) between treatment and placebo groups were obtained from the LSMEANS statement using the SAS software suite (SAS Institute, Cary, NC, USA). LSM, least-squares mean; QTcP, population-corrected QT interval.

The upper bound of the 90% CIs for the ΔΔQTcP (islatravir–placebo) of islatravir 0.75 mg and islatravir 240 mg was <10 ms at the geometric mean (GM) *C*_max_ (Table 2) and at all postdose time points (Fig. 3).

**TABLE 2:** Predicted ΔΔQTcP at *C*_max_ after single-dose oral administration of islatravir and moxifloxacin^*a*,^^*b*^

Analyte	Dose (mg)	*n*	Predicted ΔΔQTcP (ms)	
			LSM	90% CI
Islatravir	0.75	28	−0.73	−3.19 to 1.73
	240	26	0.03	−2.89 to 2.96
Moxifloxacin	400	28	13.84	12.10 to 15.58

^*a*^ The two-sided 90% CI is equivalent to a one-sided upper 95% CI for islatravir and one-sided lower 95% CI for moxifloxacin.

^*b*^ *C*_max_, maximum plasma concentration; LSM, least squares mean; QTcP, population-corrected QT interval; ΔΔQTcP, placebo-corrected QTcP change from baseline.

After the administration of a single dose of moxifloxacin 400 mg, the lower bound of the two-sided 90% CI of ΔΔQTcP at the observed *C*_max_ exceeded 5 ms (Table 2). This was also observed for all after dosing time points (Fig. 3). These findings suggest that the study design was adequate to detect changes in QTcP of the magnitude necessary to evaluate the potential of islatravir to prolong QTcP.

There were no recorded instances of heart rate >100 beats/minute, PR interval >220 ms, or QRS complex >110 ms after islatravir administration at either dose. Across all time points, a small number of incidences of abnormal T wave morphology were observed after the administration of islatravir 240 mg. There were no reports of abnormal U waves.

### Pharmacokinetics

After the administration of a single oral dose of islatravir, plasma concentrations increased rapidly: a *C*_max_ of 0.03 µM was reached after a median of 30 minutes for the 0.75-mg dose, and a *C*_max_ of 3.83 µM was reached after a median of 65 minutes for the 240-mg dose (Table 3). M4 plasma peak concentrations occurred in tandem with peak concentrations of islatravir, with the overall exposure to M4 approximately threefold lower than the exposure to islatravir. At a dose equivalent to the islatravir 0.75 mg dose, M4 was only detectable up to 4 hours after the dose; therefore, data were insufficient to characterize terminal phase–related PK parameters for M4 (Table 3).

**TABLE 3:** ** **Summary statistics of islatravir, M4, and moxifloxacin PK after single-dose oral administration to healthy participants^*a*^^*,^b^*^

Analyte	Period	Dose (mg)	*n*	Geometric mean				
				*C*_max_ (µM) (%CV)	*T*_max_, median (range) (h)	AUC_0–inf_ (h·µM) (%CV)	AUC_0–24_ (h·µM) (%CV)	AUC_0–168_ (h·µM) (%CV)
Islatravir	1	0.75	28	0.0250 (33.8)	0.50 (0.48–1.00)	0.0989 (26.7)	0.0641 (26.3)	0.0903 (26.3)
	2	240	26	3.83 (35.5)	1.08 (0.50–8.00)	29.8 (26.6)	21.2 (27.5)	28.2 (26.9)
M4	1	0.75	28	0.0223 (31.9)	0.52 (0.50–1.58)	NR	NR	NR
	2	240	26	2.07 (44.5)	1.15 (0.50–4.02)	8.52 (29.6)	7.18 (31.3)	8.34 (29.6)
Moxifloxacin	1	400	14	1,790 (30.9)	NA	NA	NA	NA
	2	400	14	1,870 (17.5)	NA	NA	NA	NA
	1/2	400	28	1,830 (24.6)	NA	NA	NA	NA

^*a*^ Moxifloxacin *C*_max_ is expressed in nanogram per milliliter.

^*b*^ AUC, area under the concentration–time curve; AUC_0–24_, area under the concentration–time curve from time 0 to 24 hours; AUC_0–168_, area under the concentration–time curve from time 0 to 168 hours; AUC_0–inf_, area under the concentration–time curve from time 0 to infinity; *C*_max_, maximum plasma concentration; CV, coefficient of variation; M4, 4′-ethynyl-2-fluoro-2′deoxyinosine; NA, not applicable; NR, not reported; *T*_max_, time to reach maximum plasma concentration.

### Safety

Single doses of islatravir 0.75 mg, islatravir 240 mg, moxifloxacin 400 mg, and matched placebos were generally well tolerated. All reported adverse events (AEs) were mild or moderate. No deaths or serious AEs were reported or discontinuations due to drug-related AEs.

Across all groups, the most frequently reported treatment-related AEs (occurring in >1 participant) were headache (*n* = 6), nausea (*n* = 5), vomiting (*n* = 3), dizziness (*n* = 2), and rash (*n* = 2). Although there were decreased lymphocyte counts in the 240-mg group, none were clinically significant, and there were no reported AEs due to these abnormalities, including decreased lymphocyte counts.

## DISCUSSION

In this thorough QT study, islatravir did not prolong QTcP and did not lead to other effects on ECG parameters in healthy adults at the lower dose of islatravir 0.75 mg or the supratherapeutic dose of islatravir 240 mg. The PK profile of islatravir was consistent with profiles in previous studies (7, 8), and both doses of islatravir were generally well tolerated.

After islatravir administration, the upper limit of the two-sided 90% CI for the ΔΔQTcP did not exceed 10 ms at any time. This 10-ms threshold (based on regulatory concern) is associated with ventricular arrhythmia that can increase the risk of torsade de pointes (11). The sensitivity of the concentration-QTc assay was confirmed by the results obtained for the moxifloxacin positive control group, showing a prolongation in QTcP, as expected. Moreover, neither dose of islatravir affected other ECG parameters such as R-R interval (ventricular rate), PR interval, or QRS interval.

The PK of islatravir and its major metabolite, M4, were also assessed in the current study. The dose-normalized plasma islatravir exposures [area under the concentration–time curve from time 0 to infinity (AUC_0–inf_) and *C*_max_] obtained for doses of islatravir 0.75 mg and islatravir 240 mg were approximately dose-proportional and comparable with those found in the phase 1 single-ascending-dose PK study (7). Likewise, dose-normalized M4 exposures (AUC_0–inf_ and *C*_max_) were comparable with those observed in healthy participants in previous phase 1 studies (9). Moxifloxacin 400 mg *C*_max_ was generally similar to the *C*_max_ reported in the literature (12).

The use of the supratherapeutic 240-mg dose of islatravir resulted in substantially higher AUC from time 0 to 24 hours (AUC_0–24_; ~330-fold increase) and *C*_max_ (~150-fold increase) GMs than the 0.75-mg dose. The drug exposure attained by administering the 240-mg dose was expected to surpass any potential increases in islatravir exposure after a 60-mg dose in a clinically relevant population due to intrinsic (i.e., age and renal/hepatic impairment) or extrinsic (i.e., concomitant drug therapy) factors (13). Relative to the dose levels being evaluated (0.25 mg once daily and 2 mg once weekly), the potential effects of islatravir on the QTc interval were investigated with a margin extending well beyond the expected therapeutic concentrations. Both doses of islatravir were generally well tolerated in the current study, with no differences in AE profile between treatment arms. No new safety signals were identified.

There are some unique design elements in the trial. Two dose levels of islatravir were studied. The use of the requisite supratherapeutic dose of 240 mg addressed the need to attain exposures that are substantial multiples above the anticipated maximum therapeutic exposure (original high dose, 60 mg monthly; current high dose in ongoing clinical trials, 2 mg once weekly). The lower dose of 0.75 mg was used to address regulatory concerns that the exposure to the 240-mg dose is orders of magnitude higher than the therapeutic exposure and may not represent findings closer to therapeutic exposure. Due to the long plasma half-life of islatravir, a standard three- or four-period crossover study could not be performed easily; therefore, the two-period nested approach was taken. The 2:2:1:4 randomization of the four treatment sequences was undertaken to ensure appropriate data collection for each of the groups of interest and considered period effect.

A comprehensive evaluation of cardiac safety is required to safely conduct drug development programs and drug registration. This double-blind, partial-crossover, active- and placebo-controlled study, which incorporated the International Council for Harmonization of Technical Requirements for Pharmaceuticals for Human Use E14 guidance into the protocol, allowed for a rigorous evaluation of the impact of islatravir on the QTc interval. Most participants in the current trial were White (88.9%); however, a pooled analysis of 20 thorough QT studies showed no evidence that race affects the relationship between moxifloxacin concentration and QTc interval (14). Therefore, the findings from the current study may be generalizable to other populations.

### Conclusions

The results of the current study indicate that a supratherapeutic dose of islatravir (240 mg) does not prolong the QTc interval or adversely affect other ECG parameters. These findings support the continued development of islatravir for the treatment of HIV-1 infection without intensive ECG collection in large phase 3 trials.

## MATERIALS AND METHODS

### Study design

A randomized, double-blind, active- and placebo-controlled, parallel-nested, crossover study (protocol MK-8591-032) was conducted at a single site. The study consisted of four sequences, with two treatment periods per sequence. Participants were randomly assigned 2:2:1:4 to a treatment sequence by use of a computer-generated allocation schedule. Prior to dosing, participants were placed on Holter monitoring to collect pre-dose readings through 24 hours postdosing. Participants were domiciled during this period to facilitate the intense PK sampling and Holter monitoring. Participants returned to the unit for period 2 and resumed Holter monitoring. Participants underwent identical PK and Holter assessments for period 2, except that the 168-hour assessment included triplicate ECGs instead of Holter extraction. In crossover sequences 1 and 2, participants received either moxifloxacin 400 mg or placebo to moxifloxacin, alongside a placebo to islatravir (both 0.75 mg and 240 mg placebos in both periods; Fig. 1). In sequence 3, participants received placebos to islatravir 0.75 mg, islatravir 240 mg, and moxifloxacin in both periods. In period 1 of sequence 4, participants received a single dose of islatravir 0.75 mg in addition to placebos to islatravir 240 mg and moxifloxacin; in period 2, participants received a single dose of islatravir 240 mg in addition to placebos to islatravir 0.75 mg and moxifloxacin (Fig. 1). Study medication was administered at approximately the same time in each treatment period. There was a minimum 7-day washout between dosing in period 1 and period 2. By administering the supratherapeutic dose only during the final period, a lengthy washout period (>1 month) for islatravir 240 mg was avoided. Any lingering islatravir from a dose of 0.75 mg in the first period would not be expected to affect the PK of a 240-mg dose.

The primary objective was to evaluate the effect of islatravir on the placebo-corrected change from baseline QTc interval. PR interval, R-R interval, QRS duration, and U and T wave morphology were additionally assessed. Moxifloxacin QTc change from baseline was evaluated to confirm assay sensitivity.

### Study population

Eligible participants were aged 18–65 years; had a body mass index ≥18.5 and ≤33 kg/m^2^; and were in good health, based on medical history, physical examination, vital signs, ECGs, and laboratory safety tests. The key exclusion criteria included a QTc interval >450 ms for males and >470 ms for females, a history of cardiac risk factors, a history of clinically significant abnormalities or diseases, or a high risk of HIV infection. The full eligibility criteria are detailed in the Supplementary Material.

### ECG assessments

Continuous ECG monitoring was performed from approximately 60 minutes before dosing in all periods until 24 hours after dosing. Triplicate, 10-second, 12-lead ECGs were extracted before dosing [at approximately 20 and 10 minutes and within 5 minutes before dosing (for baseline measurements)] and at the following after dosing time points: 0.5, 1, 1.5, 2, 3, 4, 8, 12, and 24 hours. Participants were required to lie quietly in a supine position with minimal movement and minimal exposure to noise and other environmental stimuli for at least 10 minutes before and 5 minutes during the ECG extraction window.

### Pharmacokinetic assessments

Pharmacokinetic sampling for islatravir, metabolite M4, and moxifloxacin was performed before dosing and at 0.5, 1, 1.5, 2, 3, 4, 8, 12, 24, 96, and 168 hours after dosing. When blood collections occurred at the same nominal time as ECG, ECG was performed first. Plasma islatravir and M4 concentrations were determined by the use of liquid chromatography tandem mass spectrometry (LC-MS/MS), as previously described (15). The lower limit of quantification (LLOQ) for islatravir was 20.0 pg/mL, and the LLOQ for M4 was 0.5 ng/mL. For moxifloxacin, plasma concentrations were determined by the use of high-performance LC-MS/MS, and the LLOQ was 10.0 ng/mL.

For islatravir and M4, plasma PK parameters were calculated by noncompartmental analyses using Phoenix WinNonlin Professional software (version 8.1; Pharsight, Mountain View, CA, USA). *C*_max_ and time to reach maximal concentration (*T*_max_) were generated by Phoenix WinNonlin from the observed plasma concentration–time data. AUC_0–24_ and AUC from time 0 to 168 hours (AUC_0–168_) were calculated using the linear trapezoidal method for ascending concentrations and the log trapezoidal method for descending concentrations (linear-up/log-down). AUC_0–inf_ was calculated as the sum of AUC_0-last_ + *C*_est,last_/λz, where AUC_0-last_ is the AUC to the last measurable concentration, *C*_est,last_ is the estimated concentration corresponding to the time of the last measurable concentration, and λz is the apparent terminal rate constant. For moxifloxacin, *C*_max_ was generated by Phoenix WinNonlin from the observed plasma concentration–time data.

### Safety assessments

Safety was monitored throughout the trial through the evaluation of AEs, physical examinations, vital signs, additional 12-lead ECG monitoring outside of the continuous ECG monitoring, and laboratory safety tests.

### Data analysis and statistics

The adequacy of the QT correction method was examined before data analysis. Fridericia’s formula (QTcF = QT/RR^1/3^) and a population-specific correction formula (where the population correction factor was the estimated slope in the regression analysis of log[QT] versus log[R-R]) were used to calculate the QTc from ECGs and assess graphically. The QTcP was used for all analyses.

An exposure-response model was used to characterize the relationship between islatravir and moxifloxacin concentrations and ΔQTcP, as previously described (14). ΔQTcP was evaluated using a linear mixed-effects model, with fixed effects for treatment and time point and continuous effects for islatravir or moxifloxacin plasma concentration and centered baseline QTcP; a double-compound symmetry covariance structure was assumed. An estimate of the expected mean effect, upper one-sided 95% confidence limit of ΔΔQTcP for islatravir, and lower one-sided 95% confidence limit of ΔΔQTcP for moxifloxacin were computed at the observed GM *C*_max_.

PR interval, R-R interval, QRS duration, and U and T wave morphology were descriptively summarized. Counts were provided by treatment and time point for heart rate >100 beats/minute, PR interval >220 ms, and QRS interval >110 ms.

## Data Availability

The data sharing policy, including restrictions, of Merck Sharp & Dohme LLC, a subsidiary of Merck & Co., Inc., Rahway, NJ, USA, is available at http://engagezone.msd.com/ds_documentation.php. Requests for access to the clinical study data can be submitted through the Engage Zone site or via email to Data Access mailbox.

## SUPPLEMENTAL MATERIAL

Supplemental material aac.00464-24-s0001.pdf Inclusion and exclusion criteria.

ASM does not own the copyrights to Supplemental Material that may be linked to, or accessed through, an article. The authors have granted ASM a non-exclusive, world-wide license to publish the Supplemental Material files. Please contact the corresponding author directly for reuse.
