# Supplementary material for: Thorough QT/QTc study to evaluate the effect of a single supratherapeutic dose of islatravir on QTc interval prolongation in healthy adults
Source: Antimicrob Agents Chemother. 2024 Jul 2;68(8):e00464-24. doi: 10.1128/aac.00464-24 (PMC11304681; doi:10.1128/aac.00464-24)
Supplement: Supplemental material — Inclusion and exclusion criteria. [file aac.00464-24-s0001.pdf]

Supplemental Material

Inclusion criteria

As stated in the protocol, participants were eligible to be included in the study if the participant:

1. Was in good health based on medical history, physical examination, vital sign measurements, and ECG performed before to randomization.
2. Was in good health based on laboratory safety tests obtained at the screening visit and before administration of the initial dose of study drug
3. Had a BMI ≥18.5 and ≤33 kg/m^2.
4. Was male or female, from 18 years to 65 years of age inclusive, at the time of signing the informed consent
   - For male participants, no measures were needed, but contraceptive use by men was consistent with local regulations regarding the methods of contraception for those participating in clinical studies
   - A female participant was eligible to participate if she was not pregnant or breastfeeding, and ≥1 of the following conditions applied:
     - Was not a woman of child-bearing potential
     - Was a woman of child-bearing potential and was using an acceptable contraceptive method or was abstinent from heterosexual intercourse as the preferred and usual lifestyle (abstinent on a long-term and persistent basis) during the intervention period and for 21 days after the last dose of study drug. The investigator evaluated the potential for

failure of contraceptive method (ie, noncompliance nonadherence to method of contraception, recently initiated) in relation to the first dose of study drug

    - A woman of child-bearing potential was required to have a negative highly sensitive pregnancy test serum level as required by local regulations within 72 hours before the first dose of study intervention

- The investigator was responsible for review of medical history, menstrual history, and recent sexual activity to decrease the risk of including a woman with an early undetected pregnancy
    - Contraceptive use by women was consistent with local regulations regarding the methods of contraception for those participating in clinical studies

5. The participant (or legally acceptable representative) was required to provide documented informed consent/assent for the study, including for future biomedical research

Exclusion Criteria

As stated in the protocol, participants were excluded from the study if the participant:

1. Had a history of clinically significant endocrine, gastrointestinal, cardiovascular, hematological, hepatic, immunological, renal, respiratory, genitourinary, or major neurological (including stroke and chronic seizures) abnormalities or diseases. Participants with a remote history of uncomplicated medical events (eg, uncomplicated kidney stones, defined as spontaneous passage and no

recurrence in the past 5 years, or childhood asthma) were enrolled in the study at the discretion of the investigator

2. Was mentally or legally incapacitated; had significant emotional problems at the time of the screening visit or that were expected during the conduct of the study; or had a history of a clinically significant psychiatric disorder of the past 5 years. Participants who had situational depression were enrolled in the study at the discretion of the investigator
3. Had a history of cancer (malignancy) with the following exceptions:
    - Adequately treated nonmelanomatous skin carcinoma or carcinoma in situ of the cervix
    - Other malignancies which were successfully treated with appropriate follow-up and, therefore, in the opinion of the investigator and with agreement of the sponsor, was unlikely to recur for the duration of the study, (eg, malignancies which were successfully treated 10 years before the screening visit)
4. Had a history of significant multiple and/or severe allergies (eg, food, drug, latex allergy) or had had an anaphylactic reaction or significant intolerability (ie, systemic allergic reaction) to prescription or nonprescription drugs or food
5. Was positive for hepatitis B surface antigen, hepatitis C antibodies, or HIV
6. Had major surgery or donated or lost 1 unit of blood (approximately 500 mL) within 4 weeks before the screening visit

7. Was not considered low risk of HIV infection. Low risk of HIV infection was defined by all of the following within 12 months before the screening visit (based on self-report by the participant or medical history [if available]):
    - No anal or vaginal intercourse with someone with HIV-infection or with someone of unknown HIV infection status who is at increased risk of HIV infection
    - No stimulant use (cocaine [including crack], methamphetamine, or nonphysician-prescribed pharmaceutical-grade stimulants) or inhaled nitrous oxide
    - No illicit injection drug use of any kind
    - No new diagnosis of a sexually transmitted infection such as gonorrhea (GC), chlamydia, incident syphilis, or trichomoniasis (if assessment was available). This included but was not exclusive to testing performed at screening
    - No more than three different sexual partners for receptive or insertive vaginal or anal sex
    - No history of antiretroviral therapy for HIV-1 infection, including for preexposure prophylaxis or for postexposure prophylaxis. Individuals who have participated in studies of an antiretroviral therapy, including phase 1 studies, may be eligible after consultation with the sponsor
8. Was unable to refrain from or anticipated the use of any medication, including prescription and nonprescription drugs or herbal remedies beginning approximately 2 weeks (or five half-lives) before administration of the initial dose

of study drug, throughout the study (including washout intervals between treatment periods) until the poststudy visit

9. Had participated in another investigational study within 4 weeks (or five half-lives, whichever was greater) before the screening visit. The window was derived from the date of the last visit in the previous study
10. Had a QTc interval >450 ms for males and >470 ms for females, had a history of risk factors for torsade de pointes (eg, heart failure/cardiomyopathy or family history of long QT syndrome), had uncorrected hypokalemia or hypomagnesemia, was taking concomitant medications that prolong the QT/QTc interval
11. Was under the age of legal consent
12. Was a smoker and/or had used nicotine or nicotine-containing products (eg, nicotine patch and electronic cigarette) within 3 months of screening
13. Consumed more than three glasses of alcoholic beverages (one glass was approximately equivalent to beer [354 mL/12 oz], wine [118 mL/4 oz], or distilled spirits [29.5 mL/1 oz]) per day. Participants who consumed 4 glasses of alcoholic beverages per day were enrolled at the discretion of the investigator
14. Consumed excessive amounts, defined as more than six servings (one serving was approximately equivalent to 120 mg of caffeine) of coffee, tea, cola, energy drink, or other caffeinated beverage per day
15. Was a regular user of cannabis or any illicit drug or had a history of drug (including alcohol) abuse within approximately 2 years before enrollment.

Participants were required to have a negative urine drug screening result before randomization

16. Presented any concern by the investigator regarding safe participation in the study or for any other reason the investigator considered the participant inappropriate for participation in the study
17. Was or had an immediate family member (eg, spouse, parent/legal guardian, sibling, or child) who was investigational site or sponsor staff directly involved with the current study
